# Overexpression of heparanase attenuated TGF‐β‐stimulated signaling in tumor cells

**DOI:** 10.1002/2211-5463.12190

**Published:** 2017-02-11

**Authors:** Tahira Batool, Jianping Fang, Uri Barash, Aristidis Moustakas, Israel Vlodavsky, Jin‐Ping Li

**Affiliations:** ^1^ Department of Medical Biochemistry and Microbiology and SciLifeLabUniversity of UppsalaSweden; ^2^ Faculty of MedicineCancer and Vascular Biology Research Center RappaportTechnionHaifaIsrael; ^3^ Present address: GlycoNovo Technologies Co., Ltd.ShanghaiChina

**Keywords:** cancer cell, heparan sulfate, heparanase, signaling, TGF‐beta

## Abstract

Heparan sulfate (HS) mediates the activity of various growth factors including TGF‐β. Heparanase is an endo‐glucuronidase that specifically cleaves and modifies HS structure. In this study, we examined the effect of heparanase expression on TGF‐β1‐dependent signaling activities. We found that overexpression of heparanase in human tumor cells (i.e., Fadu pharyngeal carcinoma, MCF7 breast carcinoma) attenuated TGF‐β1‐stimulated Smad phosphorylation and led to a slower cell proliferation. TGF‐β1‐stimulated Akt and Erk phosphorylation was also affected in the heparanase overexpression cells. This effect involved the enzymatic activity of heparanase, as overexpression of mutant inactive heparanase did not affect TGF‐β1 signaling activity. Analysis of HS isolated from Fadu cells revealed an increase in sulfation of the HS that had a rapid turnover in cells overexpressing heparanase. It appears that the structural alterations of HS affect the ability of TGF‐β1 to signal via its receptors and elicit a growth response. Given that heparanase expression promotes tumor growth in most cancers, this finding highlights a crosstalk between heparanase, HS, and TGF‐β1 function in tumorigenesis.

**Abbreviations**
CS: chondroitin sulfate
FGF2: fibroblast growth factor 2
HS: heparan sulfate
HSPG: heparan sulfate proteoglycans
MTS: 3‐(4,5‐dimethylthiazol‐2‐yl)‐5‐(3‐carboxymethoxyphenyl)‐2‐ (4‐sulfophenyl)‐2H‐tetrazolium, inner salt.
TGF‐β1: transforming growth factor beta 1

The complex and heterogeneous heparan sulfate proteoglycans (HSPG) are ubiquitous macromolecules expressed on the cell membrane and in the extracellular matrix, having essential roles in development and homeostasis 1, 2, 3. One of the important functions of HSPG is to mediate growth factor‐stimulated cell signaling, through interaction of the heparan sulfate (HS) side chains with growth factors and their receptors 4. The biological activity of several growth factors (e.g., FGF, PDGF, VEGF) involves dual cell surface receptor systems consisting of a tyrosine kinase‐type receptor along with a HSPG coreceptor 5. The interaction of HS with growth factors is dependent on its molecular structure that is generated through a strictly regulated biosynthesis process and postsynthesis modifications. Accumulated evidence shows that HS has enormous structural diversity, expressed in a tissue/cell‐specific manner, enabling the interaction of HS with a wide spectrum of protein ligands 6.

Heparanase is an endo‐glucuronidase that modifies HS structure through cleavage of the long HS polysaccharide chains to shorter fragments. This unique mammalian enzyme is expressed at essentially nondetectable amounts in normal tissues, but is elevated in a number of pathological conditions such as cancer and inflammation 7, 8, indicating that the enzyme has important functions in pathophysiology. Our earlier studies revealed that overexpression of heparanase in mice not only led to production of fragmented HS chains but also altered HS structure 9, 10. Increased sulfation of HS in cells overexpressing heparanase promotes FGF2 binding to its receptor and formation of a ternary complex 9.

Involvement of HS in TGF‐β‐induced signaling has been reported 11, 12, 13, 14, 15; however, information regarding the HS molecular structure in TGF‐β‐stimulated cellular activity is lacking. Heparanase was found to regulate TGF‐β expression and activity in renal fibrosis, proposing a role of heparanase in the axis of HS structure and TGF‐β activity 16. In the present study, we found reduced phosphorylation of Smad, Akt, and Erk in response to TGF‐β1 stimulation of cells overexpressing heparanase. This effect is apparently not a direct function of the overexpressed heparanase protein, but is mediated by modification of the HS structure expressed in the cells. The data provide evidence displaying that increased sulfation degree in HS is not favored by TGF‐β1, highlighting, for the first time, a crosstalk between heparanase, HS, and TGF‐β1 signaling in cancer cells.

## Materials and methods

### Reagents and cell lines

Antibodies against P‐Smad2 (cat#3101) and total Smad 2/3 (cat#3102), p‐Akt (cat#9271S) and Akt (cat# 9272), P‐Erk (cat#9101) and total Erk (cat# 9107) were purchased from Cell Signaling Technology^®^ (Danvers, MA, USA); β‐actin antibody (Sc‐69879) was from Santa‐Cruz Biotechnology (Dallas, TX, USA); Recombinant human TGF‐β1(cat#100‐21) was from PeproTech (Rocky Hill, NJ, USA). Anti heparanase antibody (1453) has been described 17. The cell lines used are Fadu (human pharyngeal carcinoma), MCF7 (human breast carcinoma), and CHO (Chinese hamster ovary), described previously 18, 19, 20. The cells were either stably (Fadu and MCF7) or transiently (CHO‐K1) overexpressing human heparanase. CHO‐K1 cells stably overexpressing double mutant enzymatically inactive heparanase were described earlier 20, 21. Cells were grown in Dulbecco's modified Eagle's medium supplemented with 10% FBS and antibiotics. Cells were passed in culture no more than 2 months after being thawed from authentic stocks.

### TGF‐β1 stimulation and western blot analysis

Cells were seeded into six‐well plates at a density of 3–6 × 10^5^ cells per well in 2 mL of DMEM supplemented with 10% FBS. After 24 h, the medium was replaced by starvation medium (DMEM without FBS) for 24 h. Then the cells were changed to fresh starvation medium containing TGF‐β1. Following stimulation for 30–60 min, medium was removed and cells were washed twice with PBS before lysis in 100 μL of RIPA buffer (50 mm Tris pH 7.5, 150 mm NaCl, 1%Triton X‐100, 1% Na‐deoxycholate, 1 mm EDTA and 0.1% SDS, Protease inhibitor, 1 mm NaF, 1 mm Na_3_VO_4_). The lysate was kept on ice for 30 min followed by ultrasonication for 3 min and centrifugation for 10 min at 16 000 ***g***. The supernatants were collected and protein concentration was determined (bicinchoninic acid assay). Samples of 20 μg of total protein were separated by electrophoresis on SDS/PAGE (10%) and electroblotted onto a Nitrocellulose membrane. The membrane was probed with antibodies and the signals were developed using Super Signal West Duration Substrate (Thermo Scientific, Waltham, MA, USA) and Bio‐Rad CCD camera. The results were analyzed by image lab™ Software (Bio‐Rad, Hercules, CA, USA). The intensity of each band was normalized with that of β‐actin or total Smad, total Akt or total Erk. The average of relative intensity is a mean of two to three blots from independent cell experiments. The intensity of Mock samples without TGF‐β1 stimulation is defined as 1.

To reduce the high endogenous signaling of Erk in Fadu cells, a TGF‐β1 inhibitor (GW6604) was included in the starvation medium (final concentration of 3 μm).

### Metabolic labeling, purification, and analysis of heparan sulfate

Fadu cells (Mock and Hpa) were cultured to 95% confluence and Na^35^SO_4_ (specific activity 1500 Ci·mmol^−1^, Perkin Elmer, Waltham, MA, USA) was added to the culture (100 μCi·mL^−1^) for 24 h before harvesting. HSPG was purified from both medium and cell fractions essentially as described 22. Briefly, the cells were lysed in buffer containing 4M Urea, 1% Triton X‐100, 50 mm Tris‐HCI, pH 7.4, 0.25 m NaCl and centrifuged. The cell lysates and medium were applied to DEAE‐Sephacel columns (GE Healthcare Biosciences, Uppsala, Sweden) pre‐equilibrated with 50 mm Tris‐HCl, 0.25 m NaCl, pH 7.4. The columns were washed with 50 mm NaAc, 0.25 m NaCl, pH 4.5 and proteoglycans were eluted with 50 mm NaAc containing 2 m NaCl, pH 4.5. The eluted material was desalted on a PD‐10 column (GE Healthcare Biosciences), followed by lyophilization to dryness. The samples were treated with Chondroitinase ABC (0.1 U per sample, Seikagaku, Tokyo, Japan) to degrade chondroitin sulfate (CS). The purified HSPG was incubated in 0.5 m NaOH on ice, to obtain free HS chains. The samples (10 000 cpm) were analyzed on a Superose 12 column (GE Healthcare Biosciences) to examine chain length or on a DEAE‐Sepharcel column (1‐mL) to assess charge density. The effluent fractions from the chromatographic separation were mixed with scintillation cocktail and counted in a scintillation counter.

### Cell proliferation assay

Fadu cells were cultured in the flasks (T‐75) to 90% confluency and then changed to starvation medium for 24 h in serum‐free medium. Then, the cells were collected and seeded in 96‐well plates (10 000 cell per well) in the starvation medium in the presence of TGF‐β1 at the concentrations indicated for 24 h. Cell proliferation was determined applying the MTS assay (Promega) by measuring absorbance at 490 nm using TECAN Plate reader. The experiments were repeated two times and the results are expressed as the mean ± SE of two independent experiments (total of 10 wells).

## Results

### TGF‐β1 induced phosphorylation of Smad, Akt, and ERK is attenuated in cancer cells overexpressing heparanase

**Figure 1:**
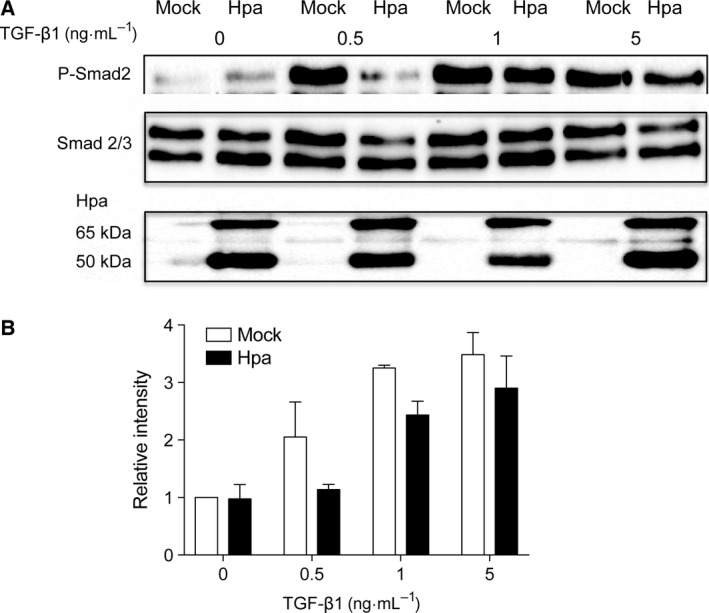
TGF‐β1‐induced Smad phosphorylation in Mock vs. Hpa Fadu cells*—*Fadu cells stably overexpressing human heparanase (Hpa) and mock (Mock) transfected cells were seeded into six‐well plates at a density of 6 × 10^5^ cells per well. After 24 h of starvation (serum‐free medium), the cells were stimulated for 30 min with TGF‐β1 at the indicated concentrations. (A) Lysate supernatants were analyzed by western blotting using anti‐phospho‐Smad2 and anti‐Smad2/3 antibodies. Overexpression of heparanase in the Hpa cells was confirmed using anti‐heparanase antibody. (B) Band intensity measured in three independent experiments was analyzed by image lab™ Software and the average band intensity of P‐Smad2 is shown. Band intensity value of Mock cells without TGF‐β1 stimulation is defined as 1.

Previous studies elucidated the involvement of heparanase in signal transduction and response to growth‐promoting factors 19, 23, 24, 25, 26. To find out the effect of heparanase expression on TGF‐β‐induced signaling, we examined phosphorylation of Smad in Fadu cells that stably overexpress human heparanase (Fig. 1A, lower panel) in comparison to mock‐transfected Fadu cells. Smad2 phosphorylation was examined by western blot analysis using an antibody that recognizes the C‐terminal diphosphorylated serine motif of Smad2 (Fig. 1A, upper panel). The results show that stimulation with TGF‐β1 led to phosphorylation of Smad2 in Fadu cells essentially in a dose‐dependent manner up to a concentration of 5 ng·mL^−1^ TGF‐β1 (Fig. 1B). Notably, cells overexpressing heparanase (Hpa) displayed a lower degree of Smad2 phosphorylation (Fig. 1A,B) as compared to mock‐transfected cells. Neither did TGF‐β1 stimulation affect expression of heparanase.

**Figure 2:**
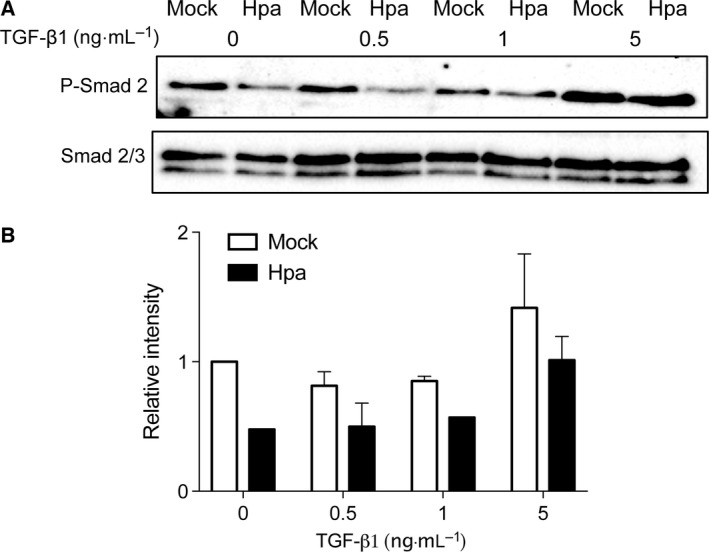
TGF‐β1‐induced Smad phosphorylation in Mock vs. Hpa MCF7 cells—cells, either stably overexpressing heparanase (Hpa) or mock transfected (Mock), were seeded into six‐well plates at a density of 6 × 10^5^ cells per well, starved and stimulated with the indicated concentrations of TGF‐β1 for 30 min. Cell lysates were analyzed by western blot (A) and quantified (B) as described in the legend to Fig. 1.

To verify whether this effect of heparanase is specific to Fadu cells, we examined additional cell lines. MCF‐7 cells showed essentially no response to TGF‐β1 at lower concentrations, irrespective of heparanase overexpression (Fig. 2). A higher dose of TGF‐β1 (5 ng·mL^−1^) stimulated Smad2 phosphorylation in both Mock and Hpa cells. Again, the Hpa cells displayed a lower degree of Smad2 phosphorylation in comparison to Mock cells. In CHO cells, the same trend was observed in heparanase high (Hpa) cells, however, the reduction in phosphorylated Smad2 was not as strong as in the human cell models (Fig. S1).

**Figure 3:**
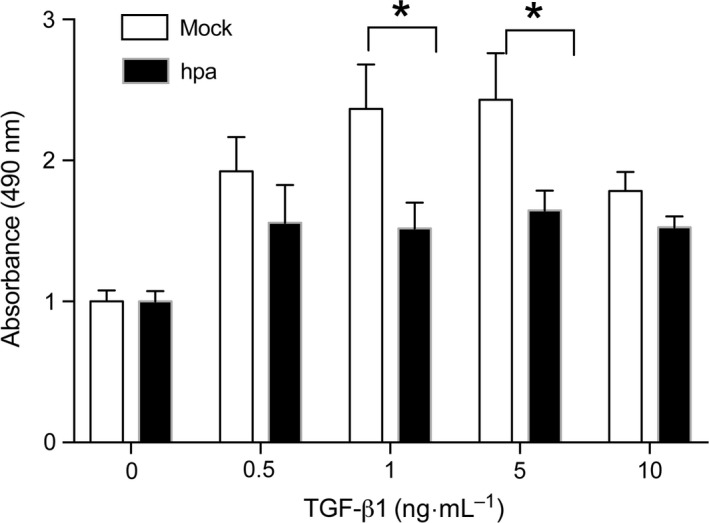
TGF‐β1‐stimulated proliferation of Fadu cells—starved Fadu cells stably overexpressing heparanase (Hpa) and mock (Mock) transfected cells were seeded on 96‐well plate (10 000 cell per well) and stimulated for 24 h with the indicated concentrations TGF‐β1. After addition of MTS reagent the absorbance at 490 nm was measured. The experiment was repeated two times and the average OD ± SE is shown. The OD of Mock cells that were not treated with TGF‐β1 is defined as 1. *P < 0.05.

To find out whether the TGF‐β1‐induced Smad phosphorylation has impact on cellular activities, we examined cell proliferation by the MTS assay. In agreement with the TGF‐β1‐stimulated phosphorylation of Smad2 (Fig. 1B), prolonged incubation (24 h) with TGF‐β1 stimulated the proliferation of Mock cells, but had no effect on Hpa cells (Fig. 3).

**Figure 4:**
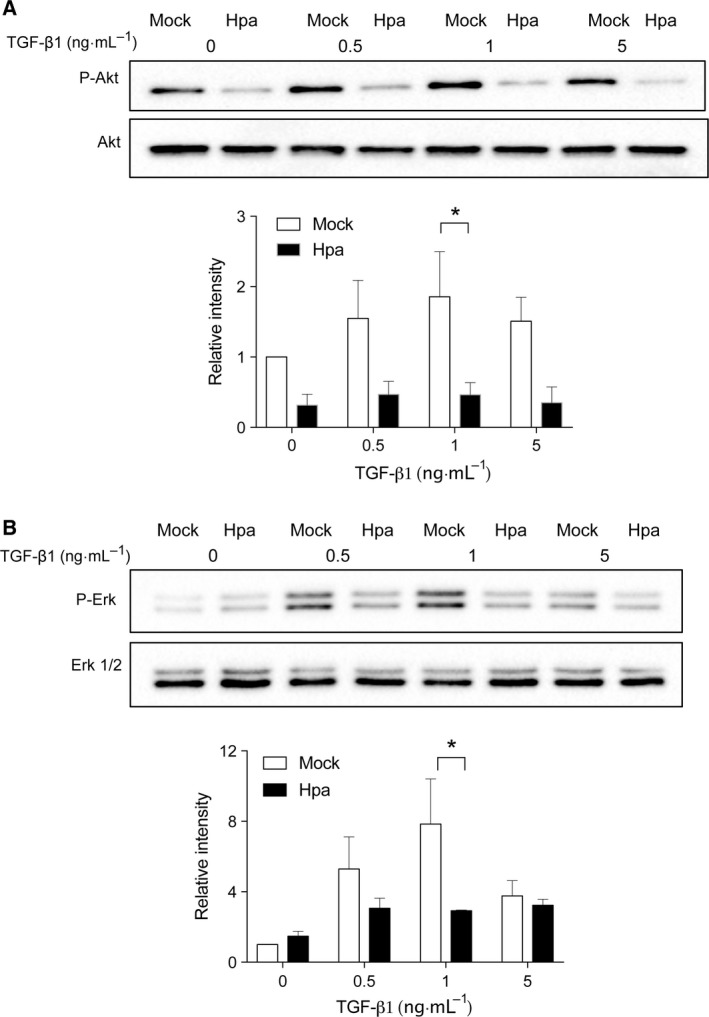
TGF‐β1‐induced Akt and Erk phosphorylation in Mock vs. Hpa Fadu cells*—*Fadu cells stably overexpressing human heparanase (Hpa) and mock (Mock) transfected cells were seeded into six‐well plates at a density of 3 × 10^5^ cells per well. After 24 h of starvation (serum‐free medium), for Akt signaling, the cells were directly stimulated for 1 h with TGF‐β1 at the indicated concentrations. For Erk, the cells were further cultured in the starvation medium in the presence of 3 μm GW6604 followed by stimulation with TGF‐β1 for 1 hr. Lysate supernatants were analyzed by western blotting using (A) anti‐phospho‐Akt and (B) anti‐Erk antibodies. Band intensity measured in three independent experiments was analyzed by image lab™ Software. The band intensity value of Mock cells without TGF‐β1 stimulation is defined as 1. *P < 0.05.

To verify if heparanase expression has a global effect on TGF‐β1‐induced signaling activity, we checked Akt and Erk signaling in the cells and found the same effect of heparanase on the TGF‐β1‐induced phosphorylation of Akt and Erk (Fig. 4). It should be noted that phosphorylation of Akt and Erk in Fadu cells is sensitive to the concentration of TGF‐β1, where 5 ng·mL^−1^ of the cytokine showed less activity than the concentrations of 0.5 and 1 ng·mL^−1^.

### Altered molecular structure of heparan sulfate in Fadu cells overexpressing heparanase

**Figure 5:**
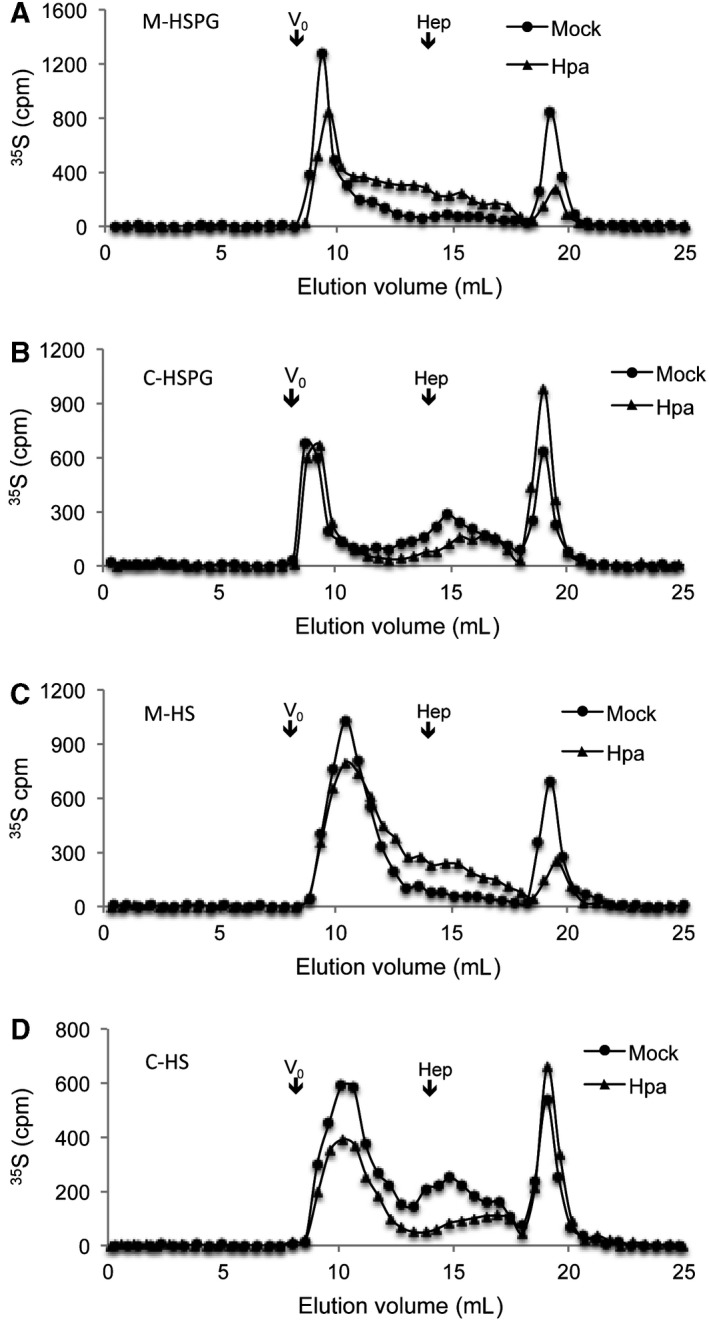
Analysis of HS chain length—Metabolically ^35^S‐labeled HS samples (10 000 cpm) from Fadu cells were separated on a Superose‐12 column showing increased amount of smaller HS fragments derived from heparanase overexpressing (Hpa) vs. Mock cells. Shown are HSPG (A, B) and HS‐free chains (C, D) from medium (A, C) and cell extracts (B, D); Vo and elution position of heparin (Hep, 14 kDa) are indicated. Degradation products (disaccharides) of CS are eluted at 18–20 mL.

To investigate whether the reduced TGF‐β1‐dependent phosphorylation in heparanase overexpressing cells is associated with the fine structure of HS, Fadu cells were cultured in the presence of ^35^S, and metabolically labeled HSPG/HS was purified. Analysis on a gel filtration chromatographic column (Superose 12) showed a marginally reduced overall molecular size of HSPG (Fig. 5A) isolated from the conditioned medium of heparanase overexpressing cells (Hpa), accompanied by elevation in the amount of smaller fragments. In the cell‐derived fractions, although the overall size of HSPG in Mock vs. Hpa cells was unchanged (Fig. 5B), the Hpa cells also displayed an accumulation of smaller fragments. This indicates an increased fragmentation of HS by overexpressed heparanase. Analysis of the free HS chains released from HSPG by alkali treatment revealed, again, accumulation of smaller fragments in both the medium (Fig. 5C) and cell (Fig. 5D) fractions. Quantification of the total ^35^S (cpm) in HS and CS fractions from the peaks showed that the ratio of HS/CS is 4.4 in the conditioned medium of Mock vs. 14.6 in the conditioned medium of Hpa‐tg Fadu cells, indicating a more than threefold increase in shedding of HS. At the same time, the HS/CS ratio was 5.4 in Mock vs. 2.7 in the Hpa high cells, indicating an increased turnover of HS in the heparanase overexpression cells (Table 1). A similar pattern of the ratio between HSPG/CSPG confirms the effect of heparanase on the intact proteoglycans.

**Table 1:** Proportion of ^35^S‐labeled HS and CS isolated from Hpa vs. Mock Fadu cells

Samples	Medium		Cell	
	Mock	Hpa	Mock	Hpa
HS/CS	4.4	14.6	5.4	2.7
HSPG/CSPG	2.3	9.9	3.2	1.6

Higher proportion of ^35^S‐labeled HSPG/HS in the Hpa medium indicates an increased shedding of the molecules. The lower proportion of HSPG/HS in the Hpa cell fractions points to a rapid turnover of the molecules.

**Figure 6:**
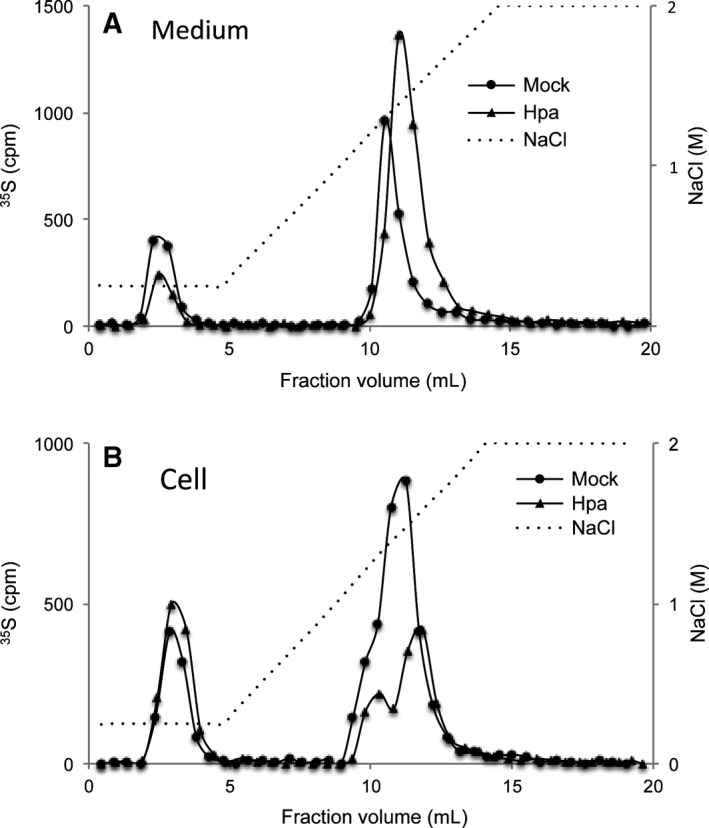
Increased overall charge density in HS from Hpa‐Fadu cells – Metabolically ^35^S‐labeled HS samples (10 000 cpm) were applied onto DEAE‐sepharcel column connected to HPLC system and eluted with a linear gradient of 0.25–2 m NaCl. The chromatograms show that HS chains derived from Hpa medium (A) and the corresponding cells (B) are more retarded in the DEAE‐sepharcel gel. The peaks eluted at low salt concentrations are degradation products of CS.

The free HS chains were further subjected to analysis on a DEAE‐Sepharcel column to evaluate the overall charge density. The degraded CS disaccharides were eluted at low salt concentrations. HS from heparanase overexpressing cells and their culture medium showed a significant retardation in the anion exchange column in comparison to the samples isolated from Mock cells, indicating a higher overall sulfation of HS from the heparanase‐overexpressing cells (Fig. 6).

### The effect of heparanase on TGF‐β1‐stimulated activity depends on its enzymatic activity

**Figure 7:**
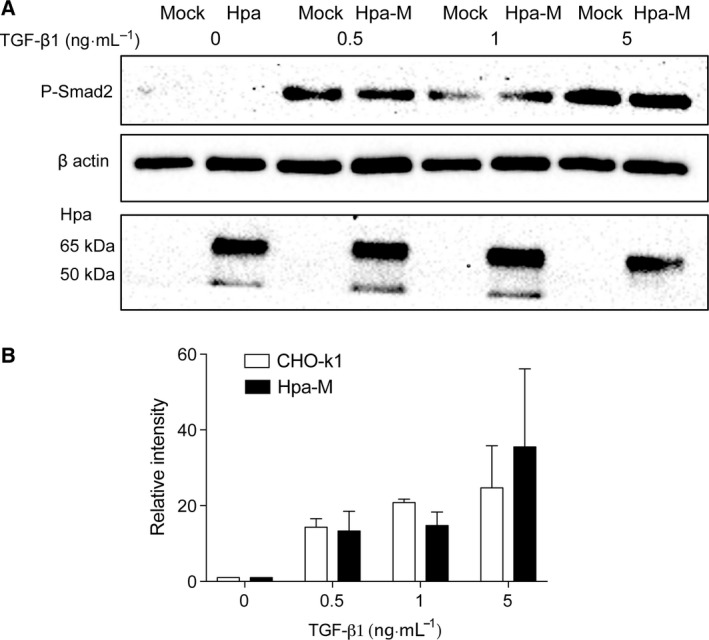
Mutant heparanase has no effect on Smad phosphorylation. CHO‐K1 cells stably overexpressing enzymatically inactive mutant human heparanase (Hpa‐M; Glu_225_, Glu_343_) or Mock transfected were seeded into six‐well plates at a density of 6 × 10^5^ cells per well. After 24 h starvation, the cells were stimulated (30 min) with TGF‐β1 at the indicated concentrations. Cell lysate supernatants were analyzed as described in the legend to Fig. 1. Cell lysates were analyzed by western blot (A) and the band intensity was quantified and presented in (B). Overexpression of mutant heparanase was confirmed using anti‐heparanase antibody (the point mutation of heparanase did not affect its epitope for recognition by the antibody) (lower panel in A).

Given that the HS structure is altered, the effect of heparanase expression on TGF‐β1‐dependent phosphorylation is presumable due to enzymatic modification of HS in the Hpa cells. To verify this, we examined Smad2 phosphorylation in CHO cell stably overexpressing mutant (Glu_225_, Glu_343_) heparanase that lacks catalytic activity 19, 21. Western blot analysis revealed that overexpression of mutant heparanase had essentially no effect on TGF‐β1‐simulated Smad2 C‐terminal phosphorylation (Fig. 7), confirming the notion that HS molecular structure plays pivotal roles in TGF‐β1 signaling activity.

## Discussion

Cell surface HS has important functional roles in formation of cytokine gradients 2 and in mediating the activities of different protein ligands including growth factors 6. The role of HS in growth factor signaling has been most studied in relation to the FGF family. In comparison, information regarding HS function in TGF‐β activity is limited. Nonetheless, several recent studies have shown that HS is involved in TGF‐β functions 11, 12, 13, 14, 15, 27. Likewise, heparanase has been shown to play a key role in renal fibrosis by regulating TGF‐β expression and activity 16. It also promotes bronchiolitis and lung fibrosis by enhancing the release and activation of ECM‐stored TGF‐β through the cleavage of HS 28. Since TGF‐β is a pluripotent cytokine that can promote or suppress cancer progression and metastasis 29, it is important to elucidate the involvement of heparanase and HS in TGF‐β‐induced signaling and cellular effects.

The HS endo‐glucuronidase (heparanase) is overexpressed in most tumor tissues, correlating with tumor vascular density and metastatic potential, and with patient survival 30. Our earlier study has found that overexpression of heparanase in mice led to production of highly sulfated HS that displayed a higher potency in assembling FGF2‐FGFR ternary structure 9. To investigate whether heparanase expression has a similar effect on TGF‐β1 activity, we examined TGF‐β1‐stimulated signaling pathways in human cancer cells that stably overexpress heparanase. Unexpectedly, we found that heparanase overexpression attenuated TGF‐β1‐induced Smad2, Akt, and Erk phosphorylation in heparanase‐overexpressing tumor cells, contradictory to its effect on FGF activity 9, 24. However, our results are in line with the finding that transgenic overexpression of an endo‐sulfatase (Sulf1) promoted activation of the TGF‐β1/Smad pathway 13. This mutual feature can be plausibly ascribed to the sulfation status of HS in the cells. Sulf1 specifically removes 6‐O‐sulfate in a given HS chain. Consequently, overexpression of sulf1 will lead to reduced HS sulfation and thereby higher TGF‐β1 activity 13. In contrast, overexpression of heparanase leads to production of highly sulfated HS (Fig. 5) 9, and hence attenuated TGF‐β1 activity.

Unlike many growth factors TGF‐β is a bifunctional regulator 31 that has both stimulatory and inhibitory activity in the same cells 32. This effect of TGF‐β is believed to be dependent on the molecular context of the cells. This feature of TGF‐β is evidenced in our experiments. Stimulation at lower concentration of TGF‐β1 increased phosphorylation of Akt and Erk in Fadu cells, but at higher concentration suppressed phosphorylation. Similarly, the cell proliferation exhibited a bell‐shape in response to the stimulation of TGF‐β1 (Figs.3, 4). Our results, together with the findings with Sulf1‐overexpressing cells 13, suggest that the molecular structure of HS on the cell surface plays a role in the pluripotent activity of TGF‐β1. Notably, HS biosynthesis and postmodification are spatially and temporally regulated by diverse factors 3, offering an explanation for the apparently contradictory activity of TGF‐β1 on the same cell type cultured under different conditions 32.

Two distinct types of FGF‐HS‐FGFR complexes were discerned; one structure showing two FGF–FGFR pairs that interact in a symmetrical mode with two oligosaccharides (2:2:2 complex) 33. The other model involves a single HS that interacts with two FGFs but only one of the two FGFR molecules (2 : 2 : 1 complex) 34. In comparison, the molecular feature of TGF‐β/HS/TGF‐β receptor interaction is largely unveiled. A model has been proposed, showing HS modulation of TGF‐β1 receptor complex formation 11. The absence of HS promotes formation of receptor Complex 1 that is constituted mostly of type II rather than type I TGF‐β1 receptor, leading to signal transduction. Thus, the attenuated TGF‐β1 activity found in the heparanase‐overexpressing cells may involve two independent or associated mechanisms; a highly charged HS on the cell surface traps TGF‐β1 preventing its interaction with receptors; or/and the increased sulfation of HS interferes with the formation of receptor Complex 1, leading to the degradation pathway 11. An additional parameter is that heparanase overexpression increases the turnover of HS, which may abrogate the stability of HS interaction with TGF‐β1, and potentially with its receptors.

In conclusion, our finding that TGF‐β1 activity is associated with the molecular structure of HS has conveyed a novel notion for the functions of TGF‐β family members. Given that heparanase expression is elevated in the majority of tumor tissues, and that HS isolated from tumor cells and tissues exhibit a higher sulfation content 9, it is of importance to establish the functions of heparanase, as well as HS, on TGF‐β1 activity and the related effects on tumor growth.

## Author contributions

TB and JPF designed and performed the experiments, wrote the manuscript. UB performed experiment IV, AM and JPL designed the study, analyzed the data, and wrote the manuscript.

## Supporting information


**Fig. S1.** CHO‐K1 cells were seeded at a density of 3 × 10^5^ cells per well of six‐well plate in 3 mL of F12K supplemented with 10% FBS and cultured for 24 h.

## References

[1] Bernfield M , Gotte M , Park PW , Reizes O , Fitzgerald ML , Lincecum J and Zako M (1999) Functions of cell surface heparan sulfate proteoglycans. Annu Rev Biochem 68, 729–777. 10872465 10.1146/annurev.biochem.68.1.729

[2] Nakato H and Li JP (2016) Functions of heparan sulfate proteoglycans in development: insights from Drosophila models. Int Rev Cell Mol Biol 325, 275–293. 27241223 10.1016/bs.ircmb.2016.02.008

[3] Li JP and Kusche‐Gullberg M (2016) Heparan sulfate: biosynthesis, structure, and function. Int Rev Cell Mol Biol 325, 215–273. 27241222 10.1016/bs.ircmb.2016.02.009

[4] Sarrazin S , Lamanna WC and Esko JD (2011) Heparan sulfate proteoglycans. Cold Spring Harb Perspect Biol 3, a004952. 21690215 10.1101/cshperspect.a004952 PMC3119907

[5] Lin X (2004) Functions of heparan sulfate proteoglycans in cell signaling during development. Development 131, 6009–6021. 15563523 10.1242/dev.01522

[6] Lindahl U and Li JP (2009) Interactions between heparan sulfate and proteins‐design and functional implications. Int Rev Cell Mol Biol 276, 105–159. 19584012 10.1016/S1937-6448(09)76003-4

[7] Vlodavsky I , Beckhove P , Lerner I , Pisano C , Meirovitz A , Ilan N and Elkin M (2012) Significance of heparanase in cancer and inflammation. Cancer Microenviron 5, 115–132. 21811836 10.1007/s12307-011-0082-7 PMC3399068

[8] Li JP and Vlodavsky I (2009) Heparin, heparan sulfate and heparanase in inflammatory reactions. Thromb Haemost 102, 823–828. 19888515 10.1160/TH09-02-0091

[9] Escobar Galvis ML , Jia J , Zhang X , Jastrebova N , Spillmann D , Gottfridsson E , van Kuppevelt TH , Zcharia E , Vlodavsky I , Lindahl U *et al* (2007) Transgenic or tumor‐induced expression of heparanase upregulates sulfation of heparan sulfate. Nat Chem Biol 3, 773–778. 17952066 10.1038/nchembio.2007.41

[10] Sandwall E , Bodevin S , Nasser NJ , Nevo E , Avivi A , Vlodavsky I and Li JP (2009) Molecular structure of heparan sulfate from Spalax: implications of heparanase and hypoxia. J Biol Chem 284, 3814–3822. 19068480 10.1074/jbc.M802196200 PMC2635051

[11] Chen CL , Huang SS and Huang JS (2006) Cellular heparan sulfate negatively modulates transforming growth factor‐beta1 (TGF‐beta1) responsiveness in epithelial cells. J Biol Chem 281, 11506–11514. 16492675 10.1074/jbc.M512821200

[12] Yue X , Lu J , Auduong L , Sides MD and Lasky JA (2013) Overexpression of Sulf2 in idiopathic pulmonary fibrosis. Glycobiology 23, 709–719. 23418199 10.1093/glycob/cwt010 PMC3641800

[13] Dhanasekaran R , Nakamura I , Hu C , Chen G , Oseini AM , Seven ES , Miamen AG , Moser CD , Zhou W , van Kuppevelt TH *et al* (2015) Activation of the transforming growth factor‐beta/SMAD transcriptional pathway underlies a novel tumor‐promoting role of sulfatase 1 in hepatocellular carcinoma. Hepatology 61, 1269–1283. 25503294 10.1002/hep.27658 PMC4376661

[14] Takashima Y , Keino‐Masu K , Yashiro H , Hara S , Suzuki T , van Kuppevelt TH , Masu M and Nagata M (2016) Heparan sulfate 6‐O‐endosulfatases, Sulf1 and Sulf2, regulate glomerular integrity by modulating growth factor signaling. Am J Physiol Renal Physiol 310, F395–F408. 26764203 10.1152/ajprenal.00445.2015

[15] Chen J , Wang Y , Chen C , Lian C , Zhou T , Gao B , Wu Z and Xu C (2016) Exogenous heparan sulfate enhances the TGF‐beta3‐induced chondrogenesis in human mesenchymal stem cells by activating TGF‐beta/Smad signaling. Stem Cells Int 2016, 1520136. 26783399 10.1155/2016/1520136 PMC4691498

[16] Masola V , Zaza G , Secchi MF , Gambaro G , Lupo A and Onisto M (2014) Heparanase is a key player in renal fibrosis by regulating TGF‐beta expression and activity. Biochim Biophys Acta 1843, 2122–2128. 24937189 10.1016/j.bbamcr.2014.06.005

[17] Zetser A , Levy‐Adam F , Kaplan V , Gingis‐Velitski S , Bashenko Y , Schubert S , Flugelman MY , Vlodavsky I and Ilan N (2004) Processing and activation of latent heparanase occurs in lysosomes. J Cell Sci 117, 2249–2258. 15126626 10.1242/jcs.01068

[18] Gross‐Cohen M , Feld S , Doweck I , Neufeld G , Hasson P , Arvatz G , Barash U , Naroditsky I , Ilan N and Vlodavsky I (2016) Heparanase 2 attenuates head and neck tumor vascularity and growth. Cancer Res 76, 2791–2801. 27013193 10.1158/0008-5472.CAN-15-1975 PMC4873389

[19] Cohen‐Kaplan V , Doweck I , Naroditsky I , Vlodavsky I and Ilan N (2008) Heparanase augments epidermal growth factor receptor phosphorylation: correlation with head and neck tumor progression. Cancer Res 68, 10077–10085. 19074873 10.1158/0008-5472.CAN-08-2910 PMC2682916

[20] Fux L , Feibish N , Cohen‐Kaplan V , Gingis‐Velitski S , Feld S , Geffen C , Vlodavsky I and Ilan N (2009) Structure‐function approach identifies a COOH‐terminal domain that mediates heparanase signaling. Cancer Res 69, 1758–1767. 19244131 10.1158/0008-5472.CAN-08-1837 PMC2650747

[21] Hulett MD , Hornby JR , Ohms SJ , Zuegg J , Freeman C , Gready JE and Parish CR (2000) Identification of active‐site residues of the pro‐metastatic endoglycosidase heparanase. Biochemistry 39, 15659–15667. 11123890 10.1021/bi002080p

[22] Jia J , Maccarana M , Zhang X , Bespalov M , Lindahl U and Li JP (2009) Lack of L‐iduronic acid in heparan sulfate affects interaction with growth factors and cell signaling. J Biol Chem 284, 15942–15950. 19336402 10.1074/jbc.M809577200 PMC2708889

[23] Fux L , Ilan N , Sanderson RD and Vlodavsky I (2009) Heparanase: busy at the cell surface. Trends Biochem Sci 34, 511–519. 19733083 10.1016/j.tibs.2009.06.005 PMC2755511

[24] Reiland J , Kempf D , Roy M , Denkins Y and Marchetti D (2006) FGF2 binding, signaling, and angiogenesis are modulated by heparanase in metastatic melanoma cells. Neoplasia 8, 596–606. 16867222 10.1593/neo.06244 PMC1601937

[25] Riaz A , Ilan N , Vlodavsky I , Li JP and Johansson S (2013) Characterization of heparanase‐induced phosphatidylinositol 3‐kinase‐AKT activation and its integrin dependence. J Biol Chem 288, 12366–12375. 23504323 10.1074/jbc.M112.435172 PMC3636920

[26] Cohen‐Kaplan V , Jrbashyan J , Yanir Y , Naroditsky I , Ben‐Izhak O , Ilan N , Doweck I and Vlodavsky I (2012) Heparanase induces signal transducer and activator of transcription (STAT) protein phosphorylation: preclinical and clinical significance in head and neck cancer. J Biol Chem 287, 6668–6678. 22194600 10.1074/jbc.M111.271346 PMC3307274

[27] Chung SW , Kwon MY , Kang YH , Chung HT , Lee SJ , Kim HP and Perrella MA (2012) Transforming growth factor‐beta1 suppression of endotoxin‐induced heme oxygenase‐1 in macrophages involves activation of Smad2 and downregulation of Ets‐2. J Cell Physiol 227, 351–360. 21437904 10.1002/jcp.22741 PMC3132305

[28] He L , Sun F , Wang Y , Zhu J , Fang J , Zhang S , Yu Q , Gong Q , Ren B , Xiang X *et al* (2016) HMGB1 exacerbates bronchiolitis obliterans syndrome via RAGE/NF‐kappaB/HPSE signaling to enhance latent TGF‐beta release from ECM. Am J Transl Res 8, 1971–1984. 27347307 PMC4891412

[29] Bierie B and Moses HL (2006) TGF‐beta and cancer. Cytokine Growth Factor Rev 17, 29–40. 16289860 10.1016/j.cytogfr.2005.09.006

[30] Vlodavsky I , Goldshmidt O , Zcharia E , Atzmon R , Rangini‐Guatta Z , Elkin M , Peretz T and Friedmann Y (2002) Mammalian heparanase: involvement in cancer metastasis, angiogenesis and normal development. Semin Cancer Biol 12, 121–129. 12027584 10.1006/scbi.2001.0420

[31] Heldin CH and Moustakas A (2016) Signaling receptors for TGF‐beta family members. Cold Spring Harb Perspect Biol. doi: 10.1101/cshperspect.a022053. 10.1101/cshperspect.a022053 PMC4968163 27481709

[32] Roberts AB and Sporn MB (1985) Transforming growth factors. Cancer Surv 4, 683–705. 2890434

[33] Schlessinger J , Plotnikov AN , Ibrahimi OA , Eliseenkova AV , Yeh BK , Yayon A , Linhardt RJ and Mohammadi M (2000) Crystal structure of a ternary FGF‐FGFR‐heparin complex reveals a dual role for heparin in FGFR binding and dimerization. Mol Cell 6, 743–750. 11030354 10.1016/s1097-2765(00)00073-3

[34] Pellegrini L , Burke DF , von Delft F , Mulloy B and Blundell TL (2000) Crystal structure of fibroblast growth factor receptor ectodomain bound to ligand and heparin. Nature 407, 1029–1034. 11069186 10.1038/35039551

