# Supplementary figures and images for: Overexpression of heparanase attenuated TGF‐β‐stimulated signaling in tumor cells
Source: FEBS Open Bio. 2017 Feb 11;7(3):405–13. doi: 10.1002/2211-5463.12190 (PMC5337900; doi:10.1002/2211-5463.12190)

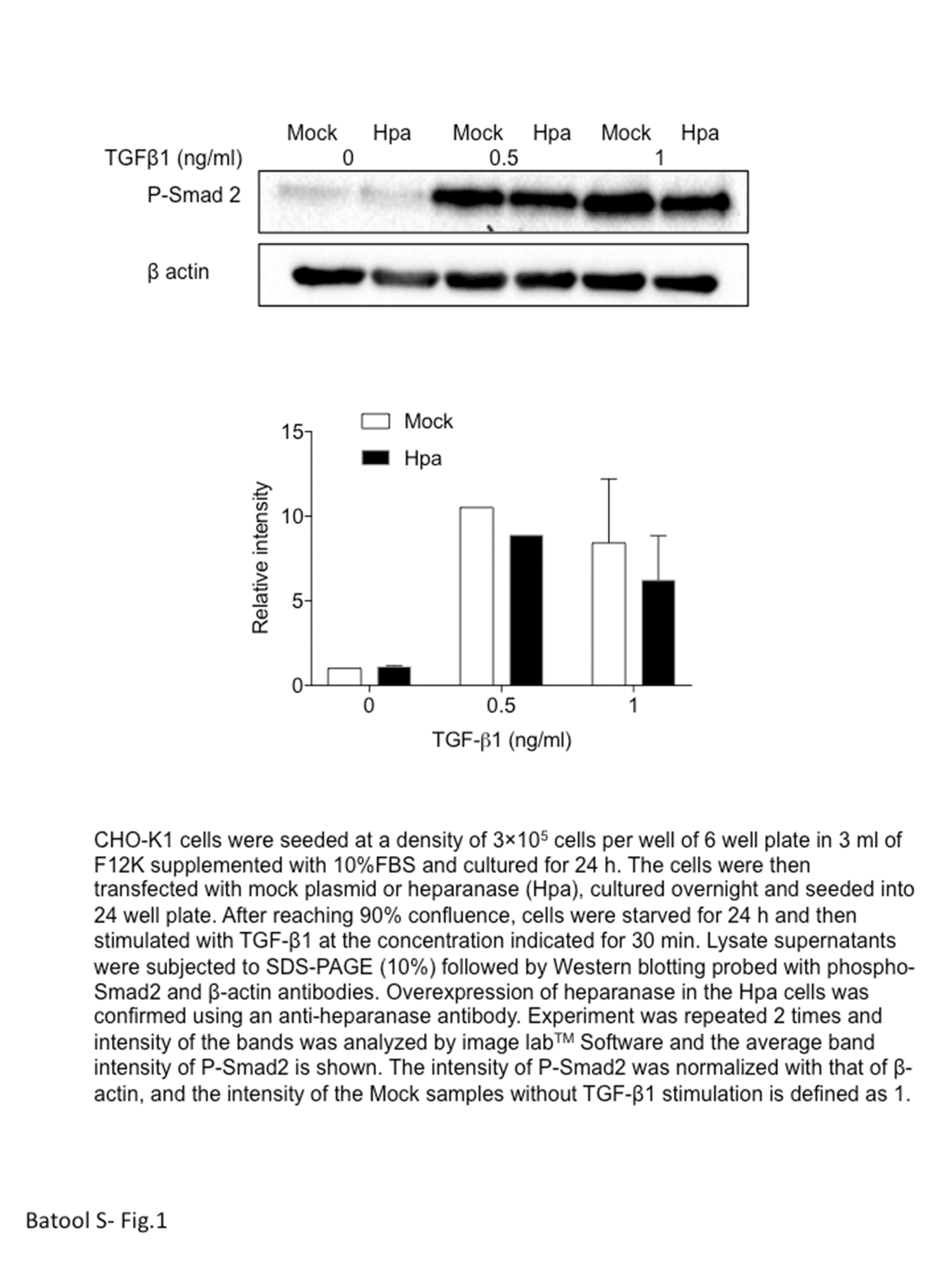

Supplement: Supplementary file 1 — Fig. S1. CHO‐K1 cells were seeded at a density of 3 × 10^5^ cells per well of six‐well plate in 3 mL of F12K supplemented with 10% FBS and cultured for 24 h. [file FEB4-7-405-s001.tif]
